# Systems pharmacology in combination with proteomics reveals underlying mechanisms of Xihuang pill against triple-negative breast cancer

**DOI:** 10.1080/21655979.2020.1834726

**Published:** 2020-10-22

**Authors:** Xingchao Xu, Jimei Zhang, Zhenhua Zhang, Meng Wang, Yaping Liu, Xiangqi Li

**Affiliations:** a Department of Breast Surgery, The Second Affiliated Hospital of Shandong First Medical University, Tai’an, China; b School of Pharmacy, Shandong First Medical University & Shandong Academy of Medical Sciences, Tai’an, China; c Department of Graduate Student Affairs, Shandong First Medical University & Shandong Academy of Medical Sciences, Tai’an, China

**Keywords:** Triple-negative breast cancer, traditional Chinese medicine, Xihuang pill, systems pharmacology, proteomics

## Abstract

Xihuang pill (XHP), a traditional Chinese herbal formula, has been clinically used as an adjuvant therapy against triple-negative breast cancer (TNBC) via inhibiting cancer cell invasion and proliferation, as well as promoting cancer cell apoptosis. However, its anti-TNBC bio-active ingredients and possible mechanisms are still unclear. Herein, the hub bio-active compounds and underlying mechanisms of XHP against TNBC were systematically elucidated by integrating systems pharmacology approach and *in vitro* proteomics analysis. Using systems pharmacology analysis and molecular docking evaluation, 28 bio-active compounds and 10 potential therapeutic targets of XHP were identified. Functional analysis showed that the core therapeutic targets against TNBC were mainly involved in epidermal growth factor receptor (EGFR)-phosphatidylinositol 3-kinase (PI3K)-AKT signaling pathway to prevent cancer cell proliferation and angiogenesis, as well as to enhance cancer cell apoptosis. The *in vitro* proteomics analysis identified 153 differentially expressed proteins (DEPs), including HASP90AA1, AKT1, and EGFR, which were also identified as therapeutic targets against TNBC through systems pharmacology analysis. Protein function analysis showed that the DEPs were mainly involved in PI3K-AKT signaling pathway, which was consistent with the result of systems pharmacology, suggesting the reliability of systems pharmacology analysis. Taken together, these findings uncover the underlying mechanism of XHP against TNBC, and provide a scientific method for the rational development of traditional Chinese medicine.

## Graphical_abstract

**Figure:**
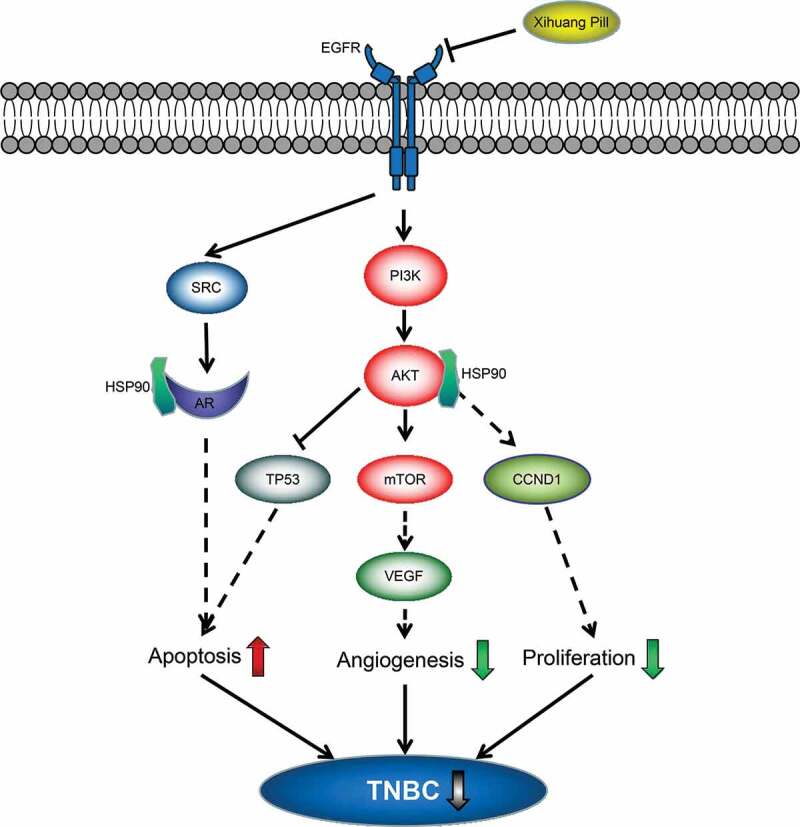

## 1. Introduction

In women, breast cancer (BC) is among the most commonly occurring cancers and the second largest cause of death worldwide after cardiovascular disease. The statistics from 2020 show approximately 276,480 women are diagnosed with BC and estimation of 42,170 women are expected to die of breast cancer [1]. Triple-negative breast cancer (TNBC) is a kind of BC that lacks estrogen and progesterone receptors as well as human epidermal growth factor receptor 2. TNBC accounts for approximately 10–15% of BC diagnoses and possesses more aggressive biological properties, with early metastatic disease, visceral metastasis, rapid disease progression, short response time to available therapies, and poor survival [2]. Owing to its aggressive behaviors, currently, there are no effective targeted therapies for TNBC except traditional chemotherapy and radiotherapy after surgery [3–6]. However, these strategies have severe side effects on patients. Therefore, it is of utmost importance to exploit a new set of strategies with high efficiency and low toxicity to prevent and treat TNBC.

In clinical practice, traditional Chinese medicine (TCM) has been widely used in China for the treatment of TNBC because of its synergistic therapeutic effects and reduced side-effects [7–10]. For instance, Huaier (*Trametes robiniophila* Murr.) granules have long been clinically used as an adjuvant therapy in post-surgical treatment for stage I–III TNBC patients, and can effectively increase the disease-free survival and overall survival of patients [11]. The rhizome of Franquet (Tubeimu) is used for treating TNBC, and its anti-metastatic effects on TNBC showed that Tubeimu can effectively inhibit the growth and metastasis of cancer cells via blocking focal adhesion pathway and changing cancer cell morphology [12]. Ai Du Qing, a clinical empirical formula, can improve the chemosensitivity of TNBC to paclitaxel through inhibiting caveolin-1 expression [13]. As a traditional Chinese herbal formula, Xihuang pill (XHP), consisting of four herbs: *Calculus bovis* (CB, Niuhuang in Chinese), *Moschus berezovskii* (MB, Shexiang in Chinese), *Resina olbani* (RO, Ruxiang in Chinese), and *Commiphora myrha* (CM, Maoyao in Chinese), has been frequently used in medical clinics for anti-TNBC therapy [14,15], and has exhibited significant effects on inhibiting proliferation and promoting apoptosis of TNBC cells [16–18]. However, information regarding core bio-active ingredients and molecular mechanism of XHP against TNBC remains to be fully explained.

In the current work, systems pharmacology approach was performed to predict the hub bio-active compounds, therapeutic targets, and possible mechanisms of XHP against TNBC, and then proteomics analysis was used to verify the result of systems pharmacology. The present study will provide a scientific reference for revealing the underlying mechanism of XHP in the treatment of TNBC.

## 2. Materials and methods

### 2.1. Systems pharmacology analysis

#### 2.1.1. Identification of bio-active compounds and related targets in XHP

The bio-active compounds of XHP were obtained from the Traditional Chinese Medicine System Pharmacology (TCMSP, http://www.tcmspw.com/tcmsp.php) database [19] and the Traditional Chinese Medicine Integrated Database (TCMID, http://www.megabionet.org/tcmid/) [20]. Drug screening criteria including oral bioavailability (OB) ≥30%, drug-like property (DL) ≥0.18, Caco-2 cell permeability (Caco-2) ≥0, and drug-like principles were applied to select the bio-active compounds [21–24]. The small molecular structure of the bio-active compounds in XHP were retrieved from the PubChem database (https://pubchem.ncbi.nlm.nih.gov/) [25]. Based on the molecular structure of the compounds, the potential targets of these compounds were screened from the online tool termed Swiss Target Prediction (http://www.swisstargetprediction.ch/) [26], and the official gene names were manually standardized using UniProtKB database (https://www.uniprot.org/) [27].

#### 2.1.2. Identification of triple-negative breast cancer (TNBC) related targets

The known TNBC-related targets were manually collected from more than 30 research papers and reviews (Table S1). These TNBC-related targets included the known targets in clinical trials, TNBC driver genes, and experimentally validated targets identified from gene knockdown or siRNA studies [28]. Subsequently, all targets obtained were manually standardized as official gene names using UniprotKB database.

#### 2.1.3. Protein-protein interaction and compounds-targets network construction

Potential therapeutic targets of XHP against TNBC were acquired via the intersection of the bio-active compounds-related targets and TNBC-related targets. These shared potential therapeutic targets were entered into STRING database (https://string-db.org/) [29] to construct protein–protein interaction (PPI) network. The PPI network with a medium confidence score >0.4 [30,31] was selected and performed to topological network analysis using the NetworkAnalyzer tool of Cytoscape v 3.7.2 software [32]. The final XHP-related key targets were acquired by meeting the value of Degree Centrality (DC), Betweenness Centrality (BC), and Closeness Centrality (CC) with its corresponding threshold value over the median value of each [33–35]. The network of core pharmaceutical bio-active compounds of XHP and key TNBC-related targets was drawn using Cytoscape software.

#### 2.1.4. GO function and KEGG pathway enrichment analyses

Gene ontology (GO) enrichment analysis and Kyoto Encyclopedia of Genes and Genomes (KEGG) pathway enrichment analysis of the core targets were performed using the Database for Annotation, Visualization and Integrated Discovery (DAVID, https://david.ncifcrf.gov/, v6.8) [36].

### 2.2. Molecular docking

Three dimensional (3D) shapes of the bio-active compounds were constructed using the ChemOffice v.17.1 software (PerkinElmer, CA, USA) and then converted into mol2 format. The 3D shapes of the core targets with PDB format were downloaded from RCSB Protein Data Bank (PDB) (http://www.rcsb.org/) [37,38]. Protein pre-processing operations including dehydrating and hydrogenation were performed using PyMOL v2.4 software (Palo Alto, CA, USA); then, the format of bio-active compounds and core targets were converted into PDBQT format using AutoDock v.4.2.6 software [39]. Subsequently, the molecular docking was performed using AutoDock Vina v. 1.1.2 software [40]. The binding energy below −20 kJ/mol was used as the screening threshold [41].

### 2.3. Proteomics analysis

#### 2.3.1. Cell culture

Human breast tumor cell MDA-MB-231 was obtained from Shanghai Institute of Biochemistry and Cell Biology (Shanghai, China). MDA-MB-231 cell was cultured in RPMI Medium 1640 (Gibco, US), supplemented with 10% of fetal bovine serum (Gibco, US) and 100 U/ml penicillin-streptomycin (Gibco, US) at 37°C and 5% CO_2_.

#### 2.3.2. Drug preparation

XHP was obtained from Tong Ren Tang Technologies Co. Ltd. (Beijing, China). A total of 5 g pills were placed in a sterile mortar and ground thoroughly into powder. Then, 3 g power was completely dissolved in 15 ml ice-cold distilled water. Subsequently, the aqueous solution was centrifuged at 5,000 × g for 20 min at 4°C. The supernatant was collected and then filtered through a 0.45 µm sterile microporous membrane, following stored at −20°C before further use. The XHP aqueous solution was diluted in RPMI Medium 1640 to desired concentrations before treatment of cancer cells.

#### 2.3.3. Protein extraction and digestion

As previously described [16,18], MDA-MB231 cells treated with 12 mg/ml XHP for 24 h were regarded as the treatment group, and the untreated cells were the control. The cells growing in the logarithmic phase were harvested, washed with cold PBS, and centrifuged at 1,500 × g for 10 min at 25°C. The cell pellets were lysed with 1 mL thiourea/urea lysis buffer containing 7 M urea, 2 M thiourea, and 0.1% (w/v) CHAPS. After completely lysed, the samples were centrifuged at 14,000 × g for 20 min at 25°C. The supernatant was recovered and quantitated by 2-D Quant Kit (Sigma-Aldrich, Oakville, ON, Canada) according to the directions. Subsequently, the proteins were reduced with 10 mM DTT (Sigma-Aldrich, Oakville, ON, Canada) for 1 h at 37°C and alkylated with 55 mM IAA (Sigma-Aldrich, Oakville, ON, Canada) for 30 min in dark condition at room temperature. After cross washing 3 times with double distilled water and acetonitrile (CAN) (Sigma-Aldrich, Oakville, ON, Canada), the proteins were digested overnight at 37°C by adding trypsin in a 1/50 ratio. Digestion was stopped with 50 μL of 2.5% trifluoroacetic acid (TFA) (Sigma-Aldrich, Oakville, ON, Canada). Peptides were retained after removing the solutions by centrifuging at 4,000 × g for 5 min at temperature. The peptides were then washed, dried, and re-solubilized in 30 μL of solution buffer containing 3% acetonitrile and 0.1% formic acid, which were employed for the following Liquid Chromatography (LC)-Mass Spectrometry (MS) analysis.

#### 2.3.4. Liquid chromatography (LC) – mass spectrometry (MS) analysis

As previously described [42], the MS analyses were conducted on a Q Exactive HF-X mass spectrometer (Thermo Fisher Scientific, Waltham, MA, USA) connected with an Easy-nLC 1000 Liquid Chromatographer (Thermo Fisher Scientific, Waltham, MA, USA). Peptides were first loaded on a commercial Acclaim PepMAP100 column (100 μm × 2 cm, C18, 5 μm) and then loaded on an EASY-Spray column (75 μm × 12 cm, C18, 3 μm). The eluants were: A. 0.1% formic acid in water and B. 0.1% formic acid in acetonitrile. The gradient was set as follows: starting at 96% A with 4% B, followed with a linear gradient from 4% to 35% B over 65 min, followed from 35–95% B over 5 min and decreased to 4% B in 3 min. Subsequently, the column was equilibrated in 4% B for 10 min. The flow rate of the column was set with 350 nL/min and the temperature was 35°C. Mass spectrometer was run in a data-dependent mode. MS scans (350–1800 m/z) were obtained at a resolution of 60,000 at m/z 400, followed by 20 data-dependent MS/MS scans. Automatic gain control values were set to 100,000 ions for survey scan and 20,000 for MS/MS scan.

#### 2.3.5. Proteomic data analysis

The raw MS/MS spectra data were searched using MaxQuant v.1.6.15 software against UniProt-SwissProt Homo sapiens canonical protein database. The N-terminal protein acetylation and methionine oxidation (15.995 Da) were set as a dynamic modification, and the cysteine carbamidomethylation (57.021 Da) was set as a static modification. A minimum peptide length was 7 amino acids and the maximum missed-cleavages were set to 2. All the enzyme specificity was set to trypsin/P. The FDR values for peptides and proteins were set to 0.01 and 0.05, respectively. Protein abundance values were estimated by normalizing the total measured spectra over all detected proteins.

### 2.4. Statistical analysis

The differential protein expression analysis was performed by a student’s t test (two-tailed) in Perseus v1.6.14.0 software and, the proteins meeting *P* ≤ 0.05 and a ratio ≥1.2 or ≤0.83 were considered as significantly expressed proteins. The core targets and protein functional enrichment analysis were conducted by a one-tail Fisher’s exact test in DAVID system and the false discovery rate (FDR) value ≤0.05 was considered as significantly enriched terms.

## 3. Results

### 3.1. Systems pharmacology analysis

#### 3.1.1. Bio-active ingredients of XHP

By the TCMSP database and TCMID screening, a total of 2,376 candidate compounds in XHP were retrieved. Based on the drug screening criteria of OB ≥30%, DL ≥0.18, Caco-2 ≥ 0, and drug-like principles, 31 nonredundant bio-active components were ultimately identified including 6 in RX, 21 in MY, 2 in SX, and 3 in NH (Table 1).

**Table 1.** The bio-active compounds of Xihuang pill (XHP)

Herb Name	Compound ID	Compound Name	MW	Hdon	Hacc	OB (%)	Caco-2	DL	Structure
RU XIANG	MOL001215	tirucallol	426.8	1	1	42.12	1.38	0.75	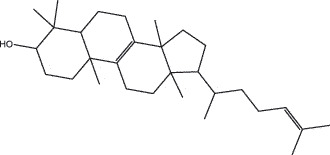
RU XIANG	MOL001243	3alpha-Hydroxy-olean-12-en-24-oic-acid	456.78	2	3	39.32	0.6	0.75	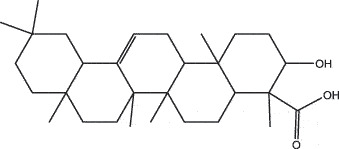
RU XIANG	MOL001255	Boswellic acid	456.78	2	3	39.55	0.59	0.75	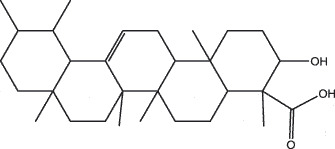
RU XIANG RU XIANG	MOL001263 MOL001265	3-oxo-tirucallic, acid acetyl-alpha-boswellic,acid	454.76 498.82	1 1	3 4	42.86 42.73	0.58 0.6	0.81 0.7	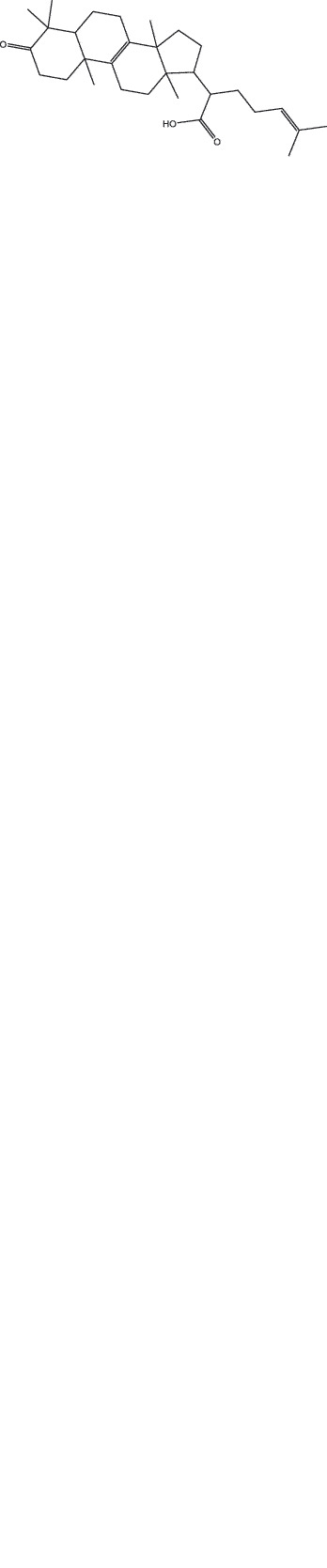 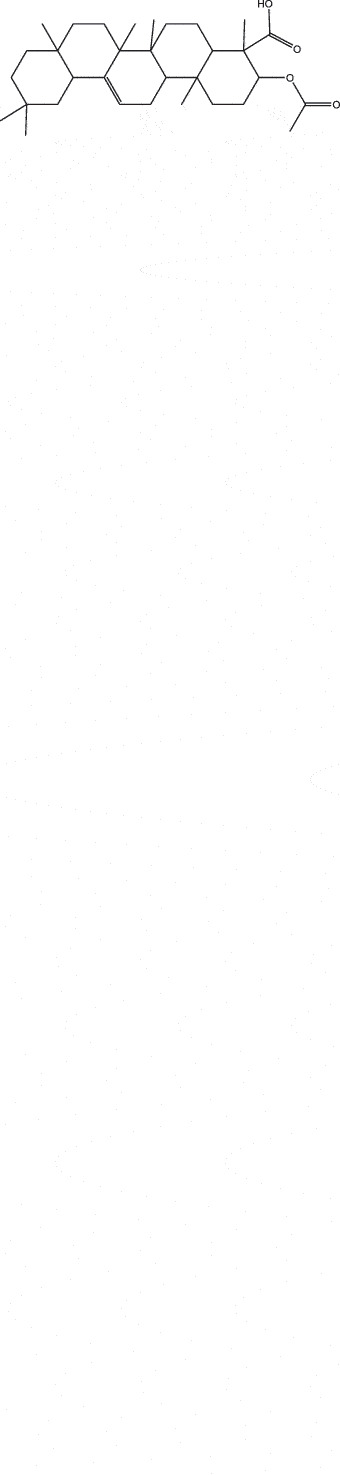
RU XIANG	MOL001272	incensole	306.54	1	2	45.59	1.33	0.22	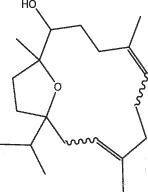
MO YAO	MOL001006	poriferasta-7,22E-dien-3beta-ol	412.77	1	1	42.98	1.45	0.76	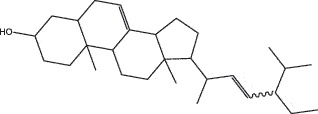
MO YAO	MOL001009	guggulsterol-VI	316.53	1	2	54.72	0.72	0.43	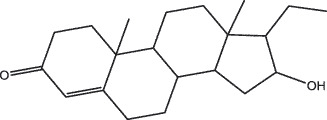
MO YAO	MOL001013	mansumbinoic acid	330.56	1	2	48.1	1	0.32	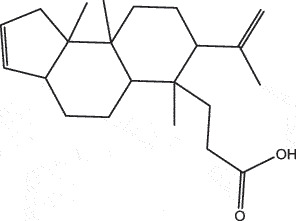
MO YAO	MOL001019	(7S,8 R,9S,10 R,13S,14S,17Z)-17-ethylidene-7-hydroxy-10,13-dimethyl-1,2,6,7,8,9,11,12,14,15-decahydrocyclopenta[a]phenanthrene-3,16-dione	328.49	1	3	35.75	0.23	0.48	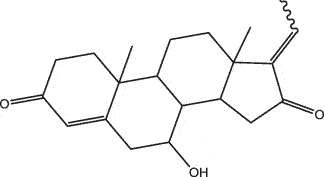
MO YAO	MOL001026	myrrhanol C	444.82	2	2	39.96	1.19	0.58	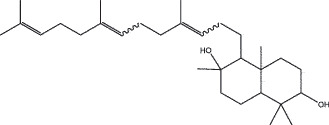
MO YAO	MOL001027	myrrhanone A	456.83	2	2	40.25	1.08	0.63	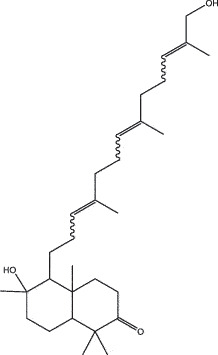
MO YAO	MOL001029	myrrhanones B	472.78	2	4	34.39	0.45	0.67	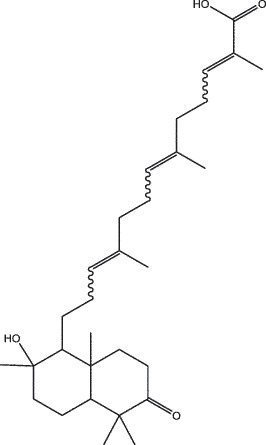
MO YAO	MOL001033	diayangambin	446.54	0	8	63.84	0.85	0.81	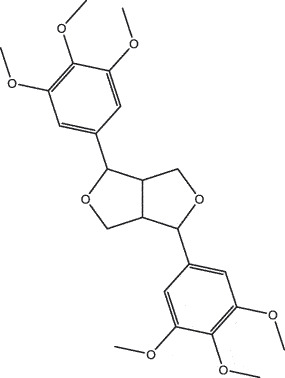
MO YAO	MOL001040	(2 R)-5,7-dihydroxy-2-(4-hydroxyphenyl)chroman-4-one	272.27	3	5	42.36	0.38	0.21	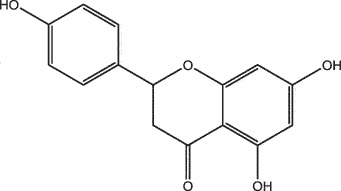
MO YAO	MOL001052	mansumbin-13(17)-en- 3,16-dione	328.54	0	2	41.78	0.78	0.45	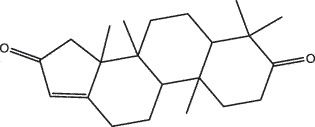
MO YAO	MOL001092	[(3R,5 R,8 R,9 R,10 R,13 R,14 R,17S)-17-[(2S,5S)-5-(2-hydroxypropan-2-yl)-2-methyloxolan-2-yl]-4,4,8,10,14-pentamethyl-2,3,5,6,7,9,11,12,13,15,16,17-dodecahydro-1H-cyclopenta[a]phenanthren-3-yl] acetate	502.86	1	4	33.07	0.69	0.8	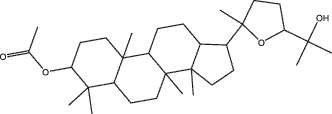
MO YAO	MOL001093	cabraleone	458.8	1	3	36.21	0.65	0.82	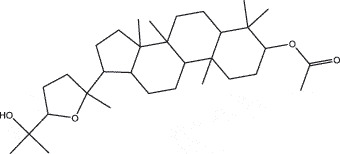
MO YAO	MOL001095	isofouquierone	456.83	1	2	40.95	0.95	0.78	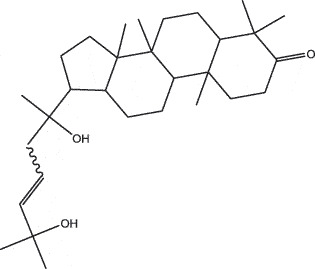
MO YAO	MOL001126	[(5aS,8aR,9 R)-8-oxo-9-(3,4,5-trimethoxyphenyl)-5,5a,6,9-tetrahydroisobenzofurano[6,5-f] [1,3]benzodioxol-8a-yl] acetate	456.48	0	9	44.08	0.6	0.9	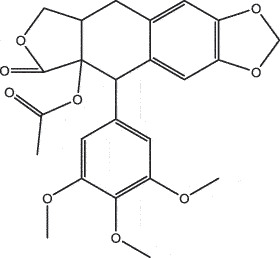
MO YAO	MOL001131	phellamurin_qt	356.4	4	6	56.6	0.14	0.39	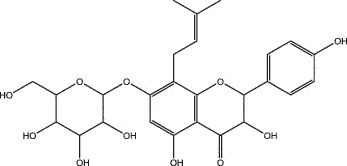
MO YAO	MOL001164	[(5S,6 R,8 R,9Z)-8-methoxy-3,6,10-trimethyl-4-oxo-6,7,8,11-tetrahydro-5H-cyclodeca[b]furan-5-yl] acetate	320.42	0	5	34.76	0.36	0.25	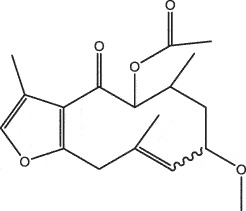
MO YAO	MOL001175	Guggulsterone	312.49	0	2	42.45	0.81	0.44	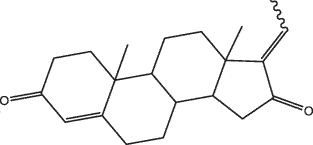
MO YAO	MOL000358	beta-sitosterol	414.79	1	1	36.91	1.32	0.75	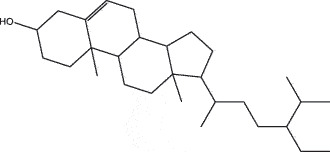
MO YAO	MOL000449	Stigmasterol	412.77	1	1	43.83	1.44	0.76	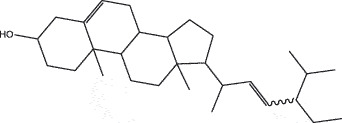
MO YAO	MOL000098	quercetin	302.25	5	7	46.43	0.05	0.28	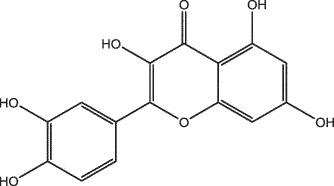
MO YAO	MOL000996	Guggulsterol IV	414.74	0	2	33.59	0.93	0.74	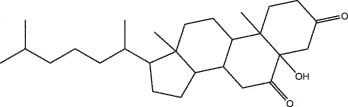
SHE XIANG	MOL000953	cholesterol	386.73	1	1	37.87	1.43	0.68	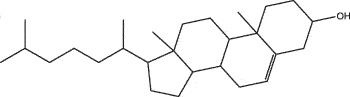
SHE XIANG	MOL000737	morin	302.25	5	7	46.23	0	0.27	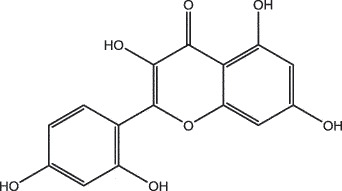
NIU HUANG	MOL008839	Methyl desoxycholate	406.67	2	4	34.63	0.25	0.73	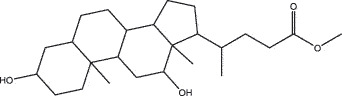
NIU HUANG	MOL008846	ZINC01280365	330.51	1	3	46.38	0.22	0.49	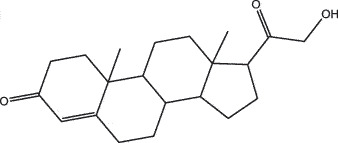
NIU HUANG	MOL000953	CLR	386.73	1	1	37.87	1.43	0.68	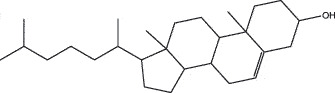

MW: molecular weight, Hdon: hydrogen-bond donors, Hacc: hydrogen-bond acceptors, OB: oral bioavailability, Caco-2: Caco-2 cell permeability, DL: drug-likeness.

#### 3.1.2. Compounds and disease-related targets screening

**Figure 1.**
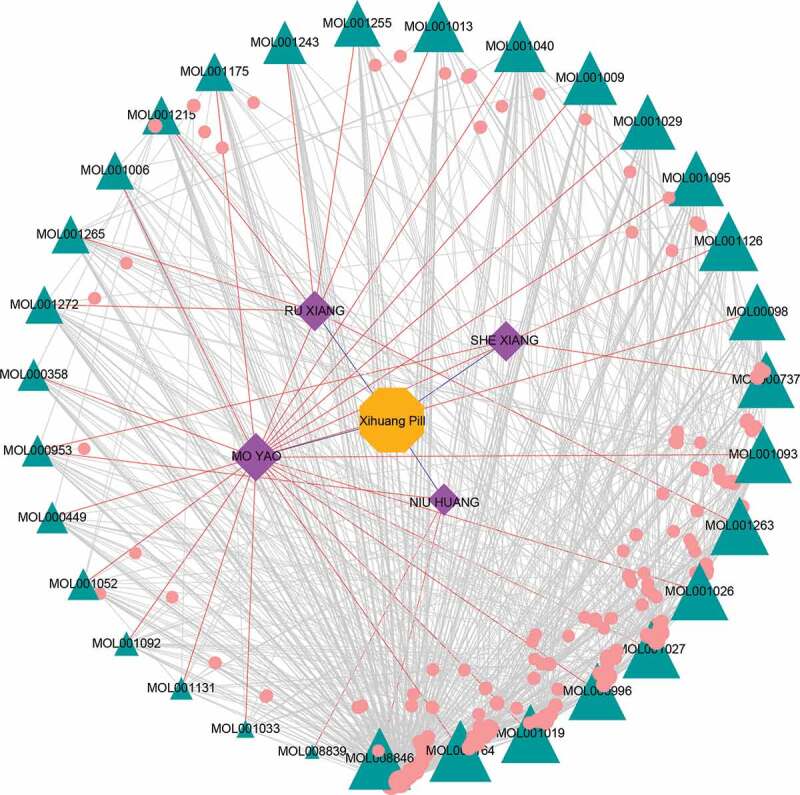
The herb-active compounds-targets network. The rhombic nodes represent the herbs in XHP, the triangle nodes represent the bio-active compounds, and the circular nodes represent the related targets of the bio-active compounds

**Figure 2.**
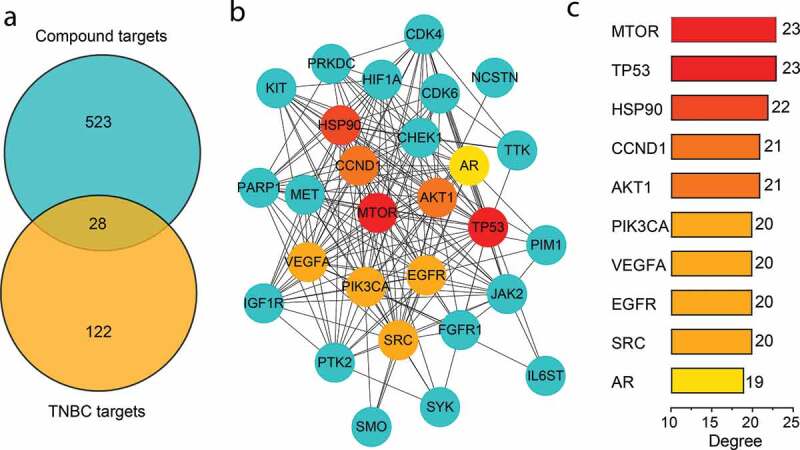
Hub targets of XHP acting on triple-negative breast cancer (TNBC). (a) Venn diagram of the related targets of bio-active compounds and TBNC; (b) Protein-protein interaction (PPI) network of 28 potential targets; (c) Hub targets in the core network

A total of 551 nonredundant compound-related targets were identified from the online tool of Swiss Target Prediction and UniProtKB databases (Figure 1, Table S2). A total of 150 potential targets related to TNBC were manually collected from more than 30 research papers and reviews (Table S1). Twenty-eight potential targets were finally acquired by calculating the intersection of the related targets of bio-active compounds and TBNC (Figure 2(a)).

#### 3.1.3. The hub targets of XHP acting on triple-negative breast cancer (TNBC)

To identify the hub targets of anti-cancer effect of XHP on TBNC, a PPI network of 28 targets were constructed by the STRING database. The PPI network was composed of 28 targets and 198 link edges based on a medium confidence score >0.4 (Figure 2(b)). According to the criteria of DC value ≥ median DC, BC value ≥ median BC, and CC value ≥ median CC of topological network analysis, the core network composed of 10 targets was obtained (Figure 2(c)).

#### 3.1.4. The bio-active compounds and hub targets network

**Figure 3.**
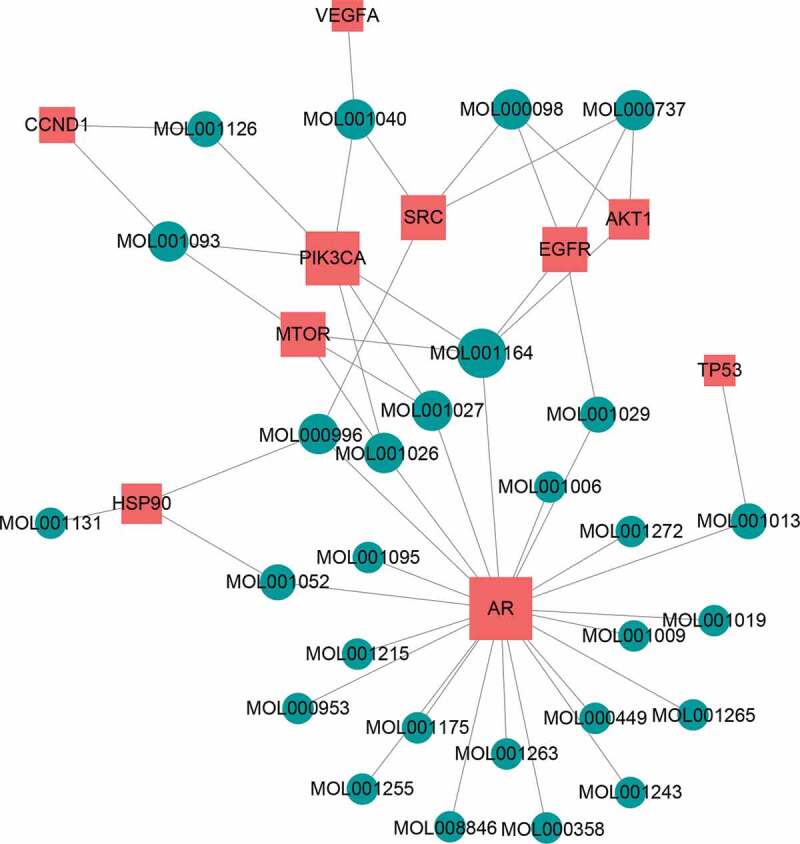
Herb-active compounds-hub targets network. Green round notes represent the herb-active compounds of XHP, and Red square nodes represent the hub targets related with TNBC. Node size represents the degree between herb-active compounds and the hub targets

To investigate the relationship between the bio-active compounds and the hub targets, the interaction network of bio-active compounds and the hub targets was established. The result showed that 28 bio-active compounds of XHP were critical to the hub targets. Among of the compounds, eight bio-active compounds linked with more than three hub targets, such as [(5S,6 R,8 R,9Z)-8-methoxy-3,6,10-trimethyl-4-oxo-6,7,8,11-tetrahydro-5H-cyclodeca[b]furan-5-yl] acetate (MOL001164, CHEMBL519180), cabraleone (MOL001093), morin (MOL000737), guggulsterol IV (MOL000996), myrrhanone A (MOL001027), myrrhanol C (MOL001026), (2 R)-5,7-dihydroxy-2-(4-hydroxyphenyl)chroman-4-one (MOL001040, naringenin), and quercetin (MOL000098) (Figure 3).

#### 3.1.5. Function enrichment analysis of hub targets

**Figure 4.**
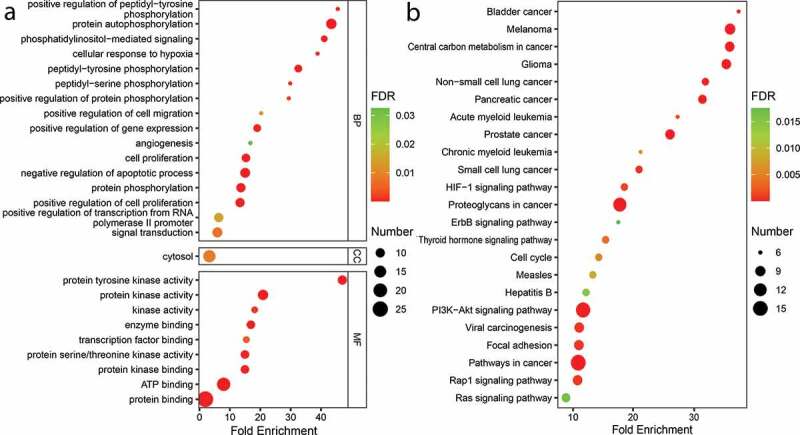
GO function and KEGG pathway enrichment analyses of the hub targets related with TNBC. (a) GO function enrichment analysis of hub targets. BP, CC and MF represent biological process, cellular component, and molecular function, respectively. (b) KEGG signaling pathway enrichment analysis of the hub targets

To understand the molecular function of the hub targets, GO analysis and KEGG pathway enrichment were conducted. GO function enrichment analysis (Figure 4(a)) showed that the hub targets were primarily distributed in molecular functions, such as kinase activity and protein binding. The key targets were also mainly involved in protein autophosphorylation, phosphatidylinositol-mediated signaling, protein phosphorylation, cell proliferation, and negative regulation of apoptotic process or cell proliferation.

KEGG pathway enrichment analysis was performed to further investigate the underlying mechanism of XHP on the treatment of TBNC. The results showed that the hub targets were highly related to PI3K-AKT signaling pathway, Rap1 signaling pathway, HIF-1 signaling pathway, focal adhesion, cell cycle, and tumor-related pathways, such as melanoma, glioma, non-small cell lung cancer, pancreatic cancer, prostate cancer, and proteoglycans in cancer (Figure 4(b)). These results indicated that XHP may have an excellent activity against TBNC, and further verified the reliability of hub targets of XHP.

### 3.2. Molecular docking evaluation

**Figure 5.**
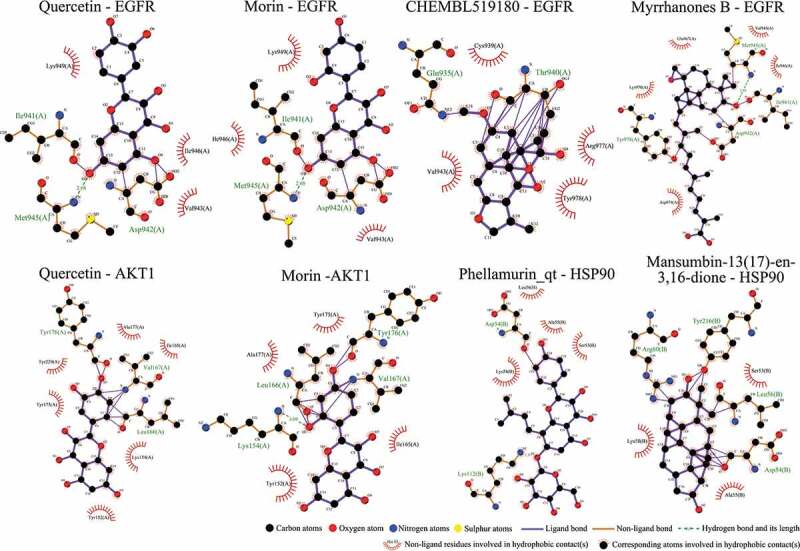
Molecular docking evaluation of the binding affinity between herb-active compounds and hub targets. The molecular docking was performed using AutoDock Vina v. 1.1.2 software with binding energy setting to −20 kJ/mol

To further confirm whether the bio-active compounds of XHP will directly interact with the hub targets, the binding affinity between compounds and hub targets was evaluated by molecular docking. The results showed that the 28 bio-active compounds of XHP had a high binding affinity with the hub targets that less than −20 kJ/mol (Table 2). For docking results, 22 compounds can directly interact with androgen receptor (AR), 7 compounds had a high binding affinity with phosphatidylinositol-4,5-bisphosphate 3-kinase catalytic subunit alpha (PIK3CA), 4 compounds interacted with epidermal growth factor receptor (EGFR), proto-oncogene, non-receptor tyrosine kinase (SRC), and mechanistic target of rapamycin kinase (MTOR), 2 compounds had binding affinity with heat-shock protein 90 alpha family class A member 1 (HSP90AA1) and serine/threonine kinase 1 (AKT1), 2 compounds interacted with cyclin D1 (CCND1), and 1 compound had a high binding affinity with tumor protein P53 (TP53) and vascular endothelial growth factor A (VEGFA). Among the compounds, for example, quercetin and morin possessed more than six binding sites with EGFR and AKT1; myrrhanones B (MOL001029) and CHEMBL51918 had at least six binding sites with EGFR; phellamurin (MOL001131) and mansumbin-13(17)-en-3,16-dione (MOL001052) had more than six binding sites with HSP90AA1 (Figure 5). This indicated that the bio-active compounds of XHP have a high binding affinity with the hub targets.

**Table 2.** The binding affinity values of the bio-active compounds and hub targets

Hub Target	Compound ID	Compound Name	Molecular Formula	Binding Affinity (kJ/mol)
AR	MOL000358	beta-sitosterol	C29H50O	−35.13
AR	MOL000449	Stigmasterol	C29H48O	−34.71
AR	MOL001095	isofouquierone	C30H50O3	−34.71
AR	MOL001215	tirucallol	C30H50O	−34.29
AR	MOL001263	3-oxo-tirucallic, acid	C30H46O3	−33.87
AR	MOL001006	poriferasta-7,22E-dien-3beta-ol	C29H48O	−33.46
AR	MOL001026	myrrhanol C	C30H52O2	−33.46
AR	MOL001029	myrrhanones B	C30H50O4	−33.46
AR	MOL001027	myrrhanone A	C30H52O3	−33.04
AR	MOL000996	Guggulsterol IV	C27H44O3	−32.62
AR	MOL000953	cholesterol	C27H46O	−32.62
AR	MOL001052	mansumbin-13(17)-en- 3,16-dione	C22H32O2	−31.78
AR	MOL001255	Boswellic acid	C30H48O3	−31.78
AR	MOL001175	Guggulsterone	C21H28O2	−31.37
AR	MOL001243	3alpha-Hydroxy-olean-12-en-24-oic-acid	C30H48O3	−31.37
AR	MOL001265	acetyl-alpha-boswellic,acid	C32H50O4	−31.37
AR	MOL001019	(7S,8 R,9S,10 R,13S,14S,17Z)-17-ethylidene-7-hydroxy-10,13-dimethyl-1,2,6,7,8,9,11,12,14,15-decahydrocyclopenta[a]phenanthrene-3,16-dione	C21H28O3	−30.95
AR	MOL001009	guggulsterol-VI	C21H32O2	−30.53
AR	MOL001013	mansumbinoic acid	C22H34O2	−28.86
AR	MOL008846	ZINC01280365	C21 H30O3	−28.44
AR	MOL001272	incensole	C20H34O2	−26.35
AR	MOL001164	[(5S,6 R,8 R,9Z)-8-methoxy-3,6,10-trimethyl-4-oxo-6,7,8,11-tetrahydro-5H-cyclodeca[b]furan-5-yl] acetate	C18H24O5	−24.26
PIK3CA	MOL001026	myrrhanol C	C30H52O2	−42.66
PIK3CA	MOL001093	cabraleone	C30H50O3	−40.57
PIK3CA	MOL001040	(2 R)-5,7-dihydroxy-2-(4-hydroxyphenyl) chroman-4-one	C15H12O5	−37.22
PIK3CA	MOL001164	[(5S,6 R,8 R,9Z)-8-methoxy-3,6,10-trimethyl-4-oxo-6,7,8,11-tetrahydro-5H-cyclodeca[b]furan-5-yl] acetate	C18H24O5	−34.71
PIK3CA	MOL001164	[(5S,6 R,8 R,9Z)-8-methoxy-3,6,10-trimethyl-4-oxo-6,7,8,11-tetrahydro-5H-cyclodeca[b]furan-5-yl] acetate	C18H24O5	−34.29
PIK3CA	MOL001126	[(5aS,8aR,9 R)-8-oxo-9-(3,4,5-trimethoxyphenyl)-5,5a,6,9-tetrahydroisobenzofurano[6,5-f] [1,3]benzodioxol-8a-yl] acetate	C24H24O9	−33.46
PIK3CA	MOL001027	myrrhanone A	C30H52O3	−30.95
EGFR	MOL001029	myrrhanones B	C30H50O4	−33.04
EGFR	MOL000098	quercetin	C15H10O7	−32.62
EGFR	MOL000737	morin	C15H10O7	−32.62
EGFR	MOL001164	[(5S,6 R,8 R,9Z)-8-methoxy-3,6,10-trimethyl-4-oxo-6,7,8,11-tetrahydro-5H-cyclodeca[b]furan-5-yl] acetate	C18H24O5	−29.27
MTOR	MOL001093	cabraleone	C30H50O3	−52.28
MTOR	MOL001027	myrrhanone A	C30H52O3	−41.40
MTOR	MOL001026	myrrhanol C	C30H52O2	−39.73
MTOR	MOL001164	[(5S,6 R,8 R,9Z)-8-methoxy-3,6,10-trimethyl-4-oxo-6,7,8,11-tetrahydro-5 H-cyclodeca[b]furan-5-yl] acetate	C18H24O5	−34.29
SRC	MOL000098	quercetin	C15 H10O7	−36.80
SRC	MOL000996	Guggulsterol IV	C27H44O3	−36.80
SRC	MOL000737	morin	C15 H10O7	−34.29
SRC	MOL001040	(2 R)-5,7-dihydroxy-2-(4-hydroxyphenyl) chroman-4-one	C15 H12O5	−33.46
AKT1	MOL000098	quercetin	C15 H10O7	−39.31
AKT1	MOL000737	morin	C15 H10O7	−38.06
CCND1	MOL001093	cabraleone	C30H50O3	−30.95
CCND1	MOL001126	[(5aS,8aR,9 R)-8-oxo-9-(3,4,5-trimethoxyphenyl)-5,5a,6,9-tetrahydroisobenzofurano[6,5-f] [1,3]benzodioxol-8a-yl] acetate	C24H24O9	−28.86
HSP90AA1	MOL001131	phellamurin_qt	C26H30O11	−38.06
HSP90AA1	MOL001052	mansumbin-13(17)-en- 3,16-dione	C22H32O2	−29.69
TP53	MOL001013	mansumbinoic acid	C22H34O2	−25.09
VEGFA	MOL001040	(2 R)-5,7-dihydroxy-2-(4-hydroxyphenyl) chroman-4-one	C15 H12O5	−31.37

### 3.3. XHP might exert anti-TNBC effects by inhibiting EGFR-PI3K-AKT signaling pathway

**Figure 6.**
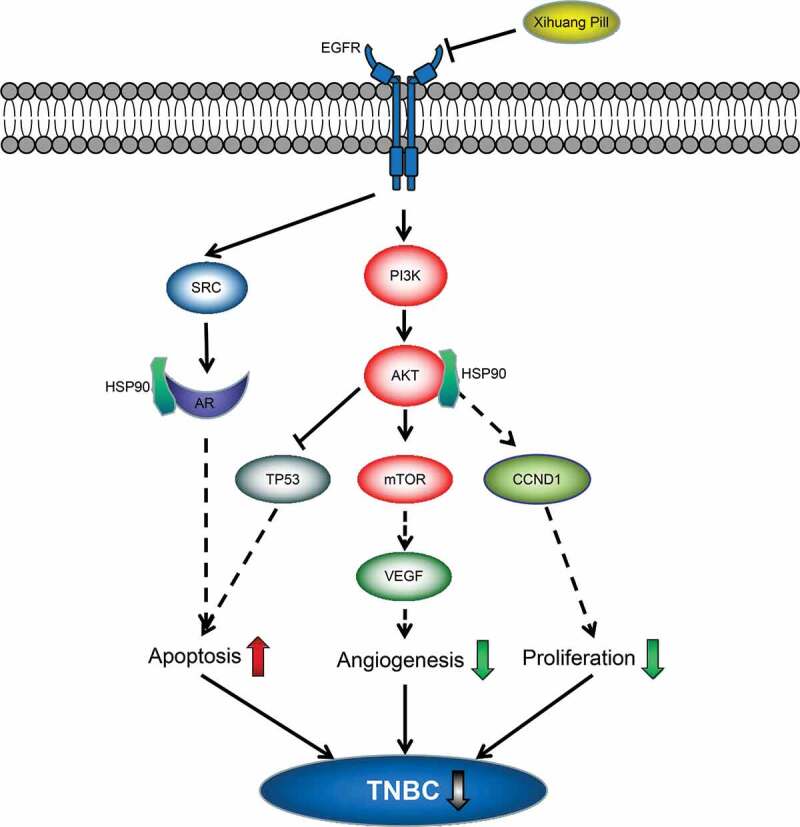
The proposed possible anti-TNBC signaling pathway of XHP. XHP might be functioned as an effective TBNC inhibitor by preventing cell proliferation, angiogenesis and enhancing apoptosis mainly through inhibiting the EGFR-PI3K-AKT signaling pathway

Based on systems pharmacology analysis and molecular docking evaluation, we proposed that XHP can exert anti-TNBC effects by preventing angiogenesis and cell proliferation and increasing apoptosis mainly through inhibiting the EGFR-phosphatidylinositol 3-kinase (PI3K)-AKT signaling pathway (Figure 6).

### 3.4. Global protein profile of normal triple-negative MDA-MB231 breast epithelial cells treated with XHP

#### 3.4.1. Protein identification and quantification

To validate the reliability of systems pharmacology, the triple-negative breast epithelial cells MDA-MB231 were treated with 12 mg/ml XHP for 24 h and following analyzed by label-free LC-MS/MS. The raw MS/MS spectra data were searched using MaxQuant against UniProt-SwissProt *Homo sapiens* canonical protein database. After quality evaluation, a total of 88,727 MS/MS spectra (135,778 matched) were harvested. Of these spectra, 18,161 peptides (16,944 unique peptides) and 2,801 protein groups (2,617 quantified proteins) were identified (Table S3).

#### 3.4.2. Identification of differentially expressed proteins

**Figure 7.**
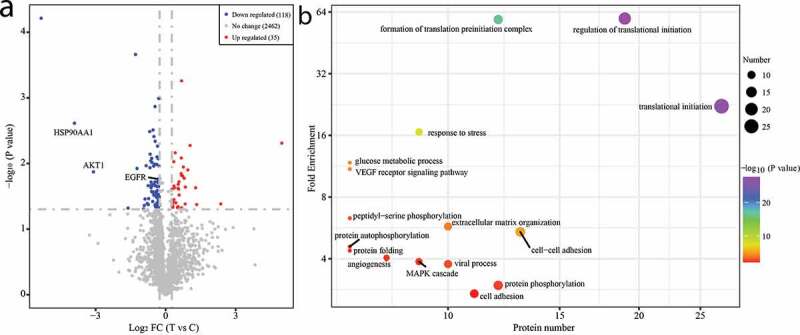
The differentially expressed proteins (DEPs) analysis. (a) Volcano plot of DEPs. (b) GO function enrichment analysis of the DEPs

To determine the differentially expressed proteins (DEPs) between XHP treated and normal triple-negative MDA-MB231 breast epithelial cells, a T test comparing analysis was performed. Based on the criterion of *P* ≤ 0.05 and a ratio ≥1.2 or ≤0.83, a total of 153 DEPs including 118 down-regulated and 35 up-regulated DEPs were obtained by comparing with normal triple-negative MDA-MB231 breast epithelial cells (Figure 7(a), Table S4). Among these DEPs, three DEPs (HASP90AA1, AKT1, and EGFR) were the shared hub targets with systems pharmacology analysis.

#### 3.4.3. Functional enrichment of DEPs

To further understand the molecular function of the DEPs, GO and KEGG pathway enrichment were performed. GO enrichment analysis showed that the DEPs were mainly involved in cell adhesion, protein phosphorylation or autophosphorylation, viral process, MAPK cascade, angiogenesis, VEGF receptor signaling pathway, glucose metabolic process, and regulation of translational initiation (Figure 7(b), Table S5).

**Figure 8.**
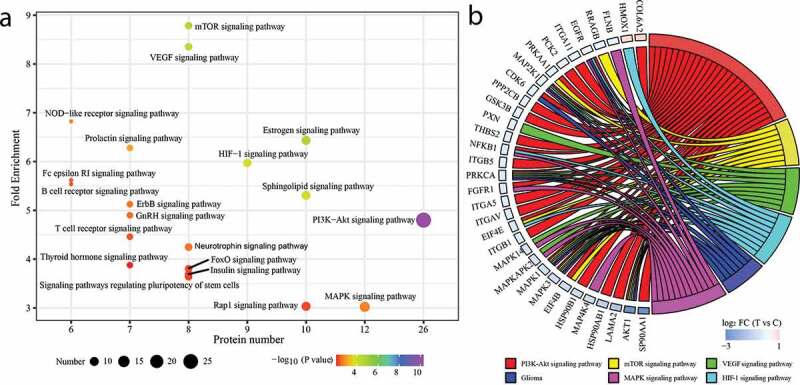
KEGG signaling pathway analysis. (a) KEGG enrichment analysis of the DEPs. (b) The circos diagram of the DEPs and its corresponding KEGG signaling pathways

KEGG pathway enrichment analysis of the DEPs showed that multiple cancer-related pathways were highly enriched, including the signaling pathways of PI3K-AKT, mitogen-activated protein kinase (MAPK), Rap1, insulin, forkhead box O (FoxO), ErbB, gonadotropin-releasing hormone (GnRH), T cell receptor, hypoxia-inducible factor-1 (HIF-1), prolactin, estrogen, VEGF, and mammalian TOR (mTOR) (Figure 8(a), Table S6). Among these pathways, PI3K-AKT and MAPK signaling pathway were the most enriched pathways, in which most of the DEPs enriched were significantly down-regulated in XHP treated triple-negative MDA-MB231 breast epithelial cells (Figure 8(b)). These results suggested that XHP can exert anti-TNBC effects through multiple targets and pathways.

## 4. Discussion

The potential bio-active ingredients and anti-TNBC mechanisms of XHP were dissected by integrating systems pharmacology and proteomics. Using systems pharmacology analysis and molecular docking evaluation, multiple bio-active compounds, and potential therapeutic targets of XHP against TNBC were identified. Meanwhile, through global protein profile of triple-negative MDA-MB231 cells treated with XHP, a large number of DEPs were identified including three intersected with systems pharmacology analysis.

Based on systems pharmacology analysis, a total of 28 bio-active compounds and 10 potential therapeutic hub targets of XHP were identified. Among these compounds, eight compounds were linked with more than three hub targets. Of these compounds, quercetin is one of the plant-derived flavonoids which is reported to possess effective anti-cancer effects on multiple molecular subtypes of breast cancer models *in vivo* or *in vitro* [43]. Many studies indicated that quercetin can induce apoptosis and cell cycle arrest and inhibit cancer cells migration and invasion in TBNC cells [44–46]. It is quercetin that was also reported to possess synergistic anticancer properties with other compounds in TBNC [47–50]. As a natural pentahydroxyflavone, morin is an anti-tumor agent and well studied to exert an antimetastatic activity in TBNC via inhibiting breast cancer cell adhesion to endothelial cells and epithelial–mesenchymal transition (EMT) [51] or suppressing highly metastatic breast cancer cells growth and invasion [52]. A recent report demonstrated that morin hydrate can effectively inhibit 12-O-tetradecanoylphorbol-13-acetate (TPA)-induced cancer cells metastatic through an Akt/GSK-3β/c-Fos signaling pathway in human MCF-7 breast cancer cells [53]. Naringenin is another naturally occurring plant flavonoid compound linked to three key targets which was found to suppress the migration and invasion of human breast cancer cells of MDA-MB-231 and MCF-7 [54–56]. In addition, the evaluation of the binding affinity between compounds and hub targets showed that these compounds had a high binding affinity with the 10 hub targets. Among the hub targets, AR is a clinically evidenced promising therapeutic target in TNBC [57–60], which can directly interact with 22 bio-active compounds. Another clinically trialed therapeutic target, PIK3CA, is the most frequent mutant gene in estrogen receptor (ER)-positive and HER2-negative advanced BC patients [61,62], which was found to link with seven compounds. Furthermore, KEGG pathway enrichment analysis of the hub targets revealed that multiple signaling pathways related to cancer were enriched. Of the signaling pathways, PI3K-AKT was the most significantly enriched signaling pathway, which was frequently activated in patients with TNBC. The pathway was considered as a promising therapeutic target, and several inhibitors targeting this pathway were designed and evaluated in various clinical trials [61,63–65]. More importantly, these findings confirmed that TCM exhibits synergistic effects against diseases through multiple-ingredient, multiple-target, and multiple-pathway approaches [9,10,23,30].

To confirm the reliability of systems pharmacology analysis, a global protein profile of TBNC MDA-MB231 cell treated with XHP was analyzed using label-free LC-MS/MS. By comparing with a normal cell, a total of 153 DEPs were obtained. KEGG function enrichment analysis showed that the DEPs were involved in several signaling pathways, including the signaling pathways of PI3K-AKT, MAPK, FoxO, VEGF, and mTOR. Of these pathways, PI3K-AKT signaling pathway was the most significantly enriched, which was consistent with the result of systems pharmacology analysis. Of note, XHP treatment can downregulate 25 different components of PI3K-AKT signaling pathway (Figure 8(b)). These effects may be of special significance given that PI3K is the most commonly frequent mutant pathway in TNBC [66,67]. The HSP90 is frequently overexpressed in TNBC patients and its elevated expression levels were found to be significantly correlated with poor prognoses [68]. It is more preclinical evidenced that HSP90 inhibition can effectively prevent cell proliferation, invasion, propagation, and angiogenesis in solid cancer by dysregulation of its client proteins, such as EGFR and AKT1 [69–73]. The EGFR level is frequently found to be overexpressed in TNBC patients and it improves aerobic glycolysis and significantly correlates with poor prognosis in TNBC patients [74–76]. The AKT1 kinase activity is found to be overexpressed in approximately 45% of primary BCs, and its silencing impairs cell proliferation and stimulates apoptosis [77]. Furthermore, caveolin-1 (CAV1), a plasma membrane protein, significantly downregulated in XHP treated group, is strongly correlated with basal-like subtype [78] and found to be significantly upregulated in inflammatory BC cells and tissues [79,80]. Another adhesion protein, desmoplakin (DSP) is significantly down-regulated by 40.31-fold in XHP treated MDA-MB-231 cells, whose expression is closely implicated in BC progression and metastasis [81]. Therefore, our DEPs analysis indicated that XHP can exert effective anti-TNBC activities via multiple targets, and further verified the reliability of systems pharmacology analysis.

In addition, several limitations exist in the present study. For example, dosage of bio-active compounds within the formula was not addressed to verify potential activity in the context of treatment as a supplement to standard therapeutic approaches, just one breast cancer cell line MDA-MB231was used as the sole method of validation of the predictions of systems pharmacology prediction, only three major DEPs were obtained through the intersection between systems pharmacology analysis and proteomics verification, and negative bio-active compounds were not selected to be subjected to systems pharmacology prediction. Therefore, it is necessary for us to address these limitations in further study.

## 5. Conclusions

Collectively, the results of systems pharmacology and proteomics indicated that XHP can effectively exert anti-TNBC activities through synergistic effects of multiple compounds, targets, and signaling pathways. We proposed that XHP can function as an effective TBNC inhibitor by preventing cancer cell proliferation and angiogenesis, as well as enhancing cancer cell apoptosis mainly through inhibiting EGFR-PI3K-AKT signaling pathway (Figure 6). Taken together, these findings uncover the underlying mechanism of XHP against TNBC and provide a scientific method for the rational development of traditional Chinese medicine.

## Supplementary Material

Supplemental Material

## Data Availability

All data generated or analyzed during this study are included in this published article (and its supplementary information files and raw data are available from the corresponding author upon reasonable request).
