# Supplementary material for: Systems pharmacology in combination with proteomics reveals underlying mechanisms of Xihuang pill against triple-negative breast cancer
Source: Bioengineered. 2020 Oct 22;11(1):1170–88. doi: 10.1080/21655979.2020.1834726 (PMC8291799; doi:10.1080/21655979.2020.1834726)
Supplement: Supplemental Material [file KBIE_A_1834726_SM1210.zip › Supplementary Materials.docx]

**Supplementary Materials**

**Table S1.** The potential targets related to triple-negative breast cancer (TNBC) by manually collecting from over 30 research articles and reviews.

**Table S2.** The herb-active compound-related targets.

**Table S3.** Peptide sequence and raw intensity.

**Table S4.** The differential expressed proteins (DEPs) identified by comparing XHP treated and normal triple-negative MDA-MB231 breast epithelial cells.

**Table S5.** GO function enrichment analysis of DEPs.

**Table S6.** KEGG signaling pathway enrichment analysis of DEPs.
